# Pathogenic Deep Intronic Variant in *CNGB3* Identified From Whole-Genome Sequencing in an Unsolved Case of Patient Affected With Achromatopsia

**DOI:** 10.1155/crig/3466358

**Published:** 2025-05-27

**Authors:** Matthew R. Gregory, Khurram Liaqat, Kayla Treat, Kathryn M. Haider, Francesco Vetrini, Erin Conboy

**Affiliations:** ^1^Indiana University School of Medicine, Riley Hospital for Children, Indianapolis, Indiana, USA; ^2^Department of Medical and Molecular Genetics, Indiana University School of Medicine, Indianapolis, Indiana, USA; ^3^Undiagnosed Rare Disease Clinic, Indiana University School of Medicine, Indianapolis, Indiana, USA; ^4^Department of Ophthalmology, Indiana University School of Medicine, Indianapolis, Indiana, USA

## Abstract

Achromatopsia (ACHM) (MIM: 262300) is an autosomal recessive disorder characterized by reduced visual acuity and color blindness. In this report, we review the case of a 14-year-old male patient diagnosed with achromatopsia with a history of retinal dystrophy, cone dysfunction with normal dark-adapted response on ERG, congenital nystagmus, farsightedness, and astigmatism. The diagnostic exome sequencing previously revealed a single maternally inherited pathogenic *CNGB3* variant (c.1148delC, p.(T383lfs∗13). Following enrollment in the Undiagnosed Rare Disease Clinic (URDC) at Indiana University School of Medicine (IUSM), genome sequencing (GS) identified a second *CNGB3* known variant c.1663-1205G > A p.(Gly555Leufs∗33), which was classified as likely pathogenic. Identification of this variant in the patient provided the evidence needed for a molecular diagnosis and ended a 15-year diagnostic odyssey for the patient and his family. With a diagnosis, the patient is eligible for gene therapy and qualifies for the state-run Vocational Rehabilitation Program and bioptic driving.

## 1. Introduction

Achromatopsia (ACHM) is a rare genetic retinal disease that is inherited in an autosomal recessive manner and estimated to affect one in 30,000 people. ACHM is characterized by presentation at birth or in early infancy with pendular nystagmus, poor visual acuity, a lack of color vision, marked photophobia, and hemeralopia [1, 2]. Cone photoreceptors are important for color vision and central visual acuity. ACHM is a clinical diagnosis where there are deficiencies in cone photoreceptors. In humans, ACHM has been classically described as a nonprogressive disease [1, 3]. It can be divided into complete or incomplete variants. Most patients with achromatopsia have complete achromatopsia. In complete achromatopsia all cone photoreceptors are nonfunctional. In incomplete achromatopsia some cone photoreceptors are at least partially functional [4].

The native cyclic nucleotide-gated (CNG) channels of rod (CNG1) and cone (CNG3) outer segments consist of α- and β-subunits. The β-subunits modulate channel properties when coexpressed with their corresponding α-subunits and thereby produce the specific characteristic of native CNG channels. Mutations in either the α-subunit (*CNGA3* gene) or the β-subunit (*CNGB3* gene) can cause achromatopsia [5, 6].

In this report, we present the case of a 14-year-old male patient who presented to the Indiana University School of Medicine Undiagnosed Rare Disease Clinic (URDC) with a history of retinal dystrophy, cone dysfunction with normal dark-adapted response on ERG, congenital nystagmus, farsightedness, astigmatism, autism spectrum disorder, attention deficit hyperactivity disorder (ADHD), and speech delay. A molecular diagnosis had not been made because clinical exome sequencing (ES) found only one pathogenic *CNGB3* variant, which was inherited from the healthy mother. The subsequent enrollment of the patient in the URDC allowed further research investigations including reanalysis of ES and GS, led to a definitive molecular diagnosis.

## 2. Case Presentation

The patient was 14 years old at the time of presentation. He was born at 36 weeks gestation, and the pregnancy was complicated by preterm labor, which was arrested with medication. There were no complications during delivery. The patient was diagnosed with congenital nystagmus and farsightedness at 3 months of age. He has worn glasses since he was 6 months old and now wears rose-tinted glasses to help improve symptoms of photophobia. He had two abnormal light adapted electroretinogram with a flat photic response and normal dark adapted response raising concern for a cone-rod dystrophy. His most recent vision testing revealed decreased visual acuity of 20/100 in both eyes. Farnsworth 15 testing showed clearly abnormal results but no clear pattern, supporting the diagnoses of color blindness. His ocular features and extensive testing were consistent with diagnosis of achromatopsia. Macular ocular coherence tomography revealed normal macular architecture. On presentation at the URDC at the age of 14, his vision has been stably low since he was five years old. Family history revealed that there were no similar vision problems in the family. No consanguinity was reported.

The patient's family enrolled the proband in the URDC at Indiana University School of Medicine (IUSM) because a prior molecular diagnosis had not been reached. A retinal dystrophy panel and ES with mitochondrial DNA were performed in patient and mother, which revealed one maternally inherited *CNGB3* pathogenic variant ((ClinVar accession: VCV000005225.70); c.1148delC p.(T383lfs∗13)). The *CNGB3* c.1148delC variant results in a frameshift, which likely causes premature termination of the protein. The patient and his mother were included in the analysis because the father was deceased. Subsequent deletion/duplication analysis targeted to the *CNGB3* gene was negative for a complete or partial deletion or duplication of *CNGB3*. Mitochondrial DNA analysis was normal. We reanalyzed the ES data obtained from the clinical sequencing laboratory and reidentified the single pathogenic *CNGB3* variant (c.1148delC p.(T383lfs∗13), maternally inherited). After URDC enrollment GS was performed on patient and mother, and results identified a second *CNGB3* deep intronic variant (c.1663-1205G > A p.(Gly555Leufs∗33), unknown inheritance). This variant was classified as likely pathogenic [7].

## 3. Discussion

In this study, we report a patient diagnosed with achromatopsia carrying the compound heterozygous variants c.1148delC p.(T383lfs∗13) and c.1663-1205G > A p.(Gly555Leufs∗33) in *CNGB3* gene. Following ES and retinal dystrophy Xpanded panel, only one pathogenic variant in *CNGB3* was found. The GS performed by URDC identified a second deep intronic variant in trans in *CNGB3* in the patient. Deep-intronic splice variants are not identified in routine exome-based diagnostics due to its focus on protein-coding regions. However, the use of novel approaches such as WGS have facilitated the scanning of entire genes and uncovered deep-intronic splice mutations in multiple retinal dystrophy genes.

The variants in the *CNGB3* gene are the major cause of achromatopsia in patients of European origin or descent [4]. The p.(Thr383Ilefs∗13) variant in *CNGB3* is the most common pathogenic variant. It accounts for over 70% of disease-causing *CNGB3* alleles and approximately 40% of all achromatopsia-associated alleles [1, 8]. Variant detection in ES is limited to exons, portions of introns, untranslated regions, intron–exon boundaries, and parts of promoters. In total, ES only analyzes about 2% of the genome. Often, this is enough to reach a definitive diagnosis. When a diagnosis cannot be reached, raw exome sequencing data can be reanalyzed, and this can lead to a definitive diagnosis [9–11]. However, many deep-intronic variants are beyond the sensitivity of ES. GS can reveal deep-intronic variants missed by ES.

This case demonstrates the utility of GS in identifying a second variant in a recessive disorder not detectable by ES in a patient apparently harboring only one disease-causing allele. For years, our patient had only one identified pathogenic *CNGB3* variant c.1148delC p.(T383lfs∗13). WGS identified the second pathogenic *CNGB3* variant c.1663-1205G > A p.(Gly555Leufs∗33). The c.1663-1205G > A variant leads to a splicing defect caused by pseudoexon insertion into the transcript [7]. Identification of presumed biallelic pathogenic variants confirms the diagnosis.

Previously, it was examined that a large cohort of achromatopsia patients harboring only a single pathogenic variant in *CNGB3* [7]. Subsequently, the sequencing of *CNGB3* in multiple unrelated achromatopsia patients identified the second *CNGB3* intronic variants in patients. Furthermore, it was also identified this deep intronic variant (c.1663-1205G > A) in 14 subjects and classified as pathogenic. Following the mini splicing assay analysis, it was found that aberrant transcript resulting from c.1663-1205G > A contains an insertion of eight amino acids followed by a premature termination codon (G555fs∗33). Even if the transcript is not targeted to nonsense mediated decay, the translated protein will lack the cGMP binding site that is essential for protein function. This variant led to a nonfunctional protein through pseudoexon activation and premature translation termination and suggested that WGS could identify a second disease-causing allele in patients with only one identified disease-causing variant [7]. Our case confirms that WGS can be used successfully in this way.

The enrollment of this patient in the URDC resolved a 15-year diagnostic odyssey. With a definitive diagnosis, the patient is eligible for gene therapy and is qualified for both the state-run Vocational Rehabilitation Program and bioptic driving. The Vocational Rehabilitation Program helps people living with disabilities to achieve their employment goals. Bioptic driving is a telescopic system used while driving that improves the sharpness of far vision. Given his diagnosis, he has been able to successfully learn to drive using bioptic driving. We explained gene therapy and the potential risks and benefits with the family, and they have decided to forgo this potential treatment at this time, as the patient is doing well and is stable. Definitive diagnoses are important for physicians and families by influencing the direction of care for a physician. For families, a diagnosis often leads to a clearer prognosis. As in this case, sometimes a diagnosis leads to an improved prognosis and treatment options, as patients with a diagnosis may qualify for programs that exclude patients without a definitive diagnosis.

## Data Availability

The data that support the findings of this study are openly available in Clinvar at https://www.ncbi.nlm.nih.gov/clinvar/, reference number VCV000005225.70 and VCV000635822.4.
